# Erkennen – Bewerten – Handeln: Wie hat der Öffentliche Gesundheitsdienst auf die COVID-19-Pandemie reagiert?

**DOI:** 10.1007/s00103-021-03302-3

**Published:** 2021-03-16

**Authors:** Viviane Bremer, Lars Schaade

**Affiliations:** 1 Abt. für Infektionsepidemiologie, Robert Koch-Institut, Seestraße 10, 13353 Berlin, Deutschland; 2 Zentrum für biologische Gefahren und spezielle Pathogene, Robert Koch-Institut, Nordufer 20, 13353 Berlin, Deutschland

Am 11.03.2020 erklärte die Weltgesundheitsorganisation (WHO) das weltweite Auftreten von SARS-CoV-2-Fällen zu einer Pandemie.^1^ Ein Jahr später ist die Pandemie noch nicht überwunden und beeinflusst das gesellschaftliche Leben nach wie vor auf vielen Ebenen. Das vorliegende Themenheft „COVID-19 und Public Health: Reaktionen des Öffentlichen Gesundheitsdienstes“ ist das zweite Heft einer vierteiligen Reihe „COVID-19 und Public Health“, die im Jahr 2021 erscheint.

Im Öffentlichen Gesundheitsdienst (ÖGD) in Deutschland wurde bereits frühzeitig das Auftreten der SARS-CoV-2-Fälle in China als mögliche Bedrohung für Deutschland erkannt [1]. Seit dem Auftreten der ersten Fälle in Deutschland ist der ÖGD in Deutschland, insbesondere die Gesundheitsämter, in einer bisher nie dagewesenen Art und Weise gefordert worden. Die Gesundheitsämter sind unter anderem verantwortlich für die Erfassung der Fälle und der dazugehörigen Kontaktpersonen sowie für die Implementierung von Isolierung und Quarantäne, die für die Unterbrechung der Transmission erforderlich sind. Diese Maßnahmen erfordern ausreichend personelle Kapazitäten in den Gesundheitsämtern. Die Landesgesundheitsbehörden unterstützen die Gesundheitsämter in ihren Aufgaben und bei der Untersuchung von Ausbruchsgeschehen. Das Robert Koch-Institut (RKI) ist in der aktuellen Pandemie zuständig für die Erfassung der aktuellen COVID-19-Lage, die Bewertung aller Informationen und die Einschätzung des Risikos für die Bevölkerung in Deutschland. Das vorliegende Themenheft beleuchtet die Reaktionen des ÖGD auf COVID-19, insbesondere, aber nicht ausschließlich aus Sicht des RKI und der Landesgesundheitsbehörden.

Erkennen, Bewerten, Handeln – diese Maxime hat sich in der Epidemiologie bewährt. Auch in der COVID-19-Pandemie bedarf es zeitnah zuverlässiger Daten, um Trends abzubilden und neue Entwicklungen zu erkennen. Auf dieser Basis muss die Situation regelmäßig bewertet werden, um die Gefährdung der Bevölkerung durch COVID-19 zuverlässig einschätzen zu können. Diese Bewertungen sind die Basis für die Formulierung von Empfehlungen für Public-Health-Maßnahmen. Entlang dieser Richtschnur sind die Beiträge in dieser Ausgabe angeordnet.

Der Beitrag von *Diercke et al.* zeigt eindrücklich, wie nach Einführung einer gesetzlichen Labormeldepflicht für SARS-CoV‑2 innerhalb kürzester Zeit das Meldesystem angepasst und ausgebaut wurde. Die hohe Anzahl der täglich gemeldeten SARS-CoV-2-Fälle und die Notwendigkeit, neu gemeldete Diagnosen täglich zu berichten, haben dazu geführt, dass die Umsetzung der elektronischen Labormeldung im Rahmen des Deutschen Elektronischen Melde- und Informationssystems für den Infektionsschutz (DEMIS; [2]) in den Gesundheitsämtern beschleunigt wurde und die Meldedaten täglich auf einem Dashboard veröffentlicht werden. Im zweiten Beitrag schildern *Goerlitz et al.*, wie die im Rahmen der Influenzapandemieplanung etablierten Überwachungssysteme an die COVID-19-Pandemie angepasst wurden. Mit den Systemen können akute respiratorische Erkrankungen (ARE) auf Bevölkerungsebene, im ambulanten und im stationären Bereich überwacht und somit die Krankheitslast von COVID-19 bzw. ARE eingeschätzt werden. Anhand dieser Systeme wurde u. a. erfasst, wie durch den ersten Lockdown im Frühjahr 2020 die Grippewelle abgekürzt werden konnte [3].

Bei der Interpretation der Fallzahlen muss bedacht werden, dass diese neben weiteren Faktoren auch von der Anzahl der durchgeführten SARS-CoV-2-Tests abhängen. Daher sollten regelmäßig Informationen zur Anzahl der durchgeführten Tests, zum Anteil der positiven Tests sowie zu den Testkapazitäten erhoben werden. Im Beitrag von *Seifried et al.* werden die am RKI durchgeführte virologische Surveillance, die wöchentlichen Abfragen der Labore zu Testaktivitäten und -kapazitäten und die Erfassung von SARS-CoV-2-Tests durch Erweiterung der Antibiotika-Resistenz-Surveillance (ARS) beschrieben. Zusätzlich zur Erfassung von Laborkapazitäten ist die zeitnahe Erfassung von Versorgungskapazitäten notwendig, um eine Überlastung der klinischen Versorgung rechtzeitig zu erkennen. *Grabenhenrich et al.* zeigen unter anderem auf, wie durch eine Automatisierung von Surveillance-Prozessen eine Echtzeit-Surveillance der Kapazitäten von Notaufnahmen und Intensivstationen aufgebaut werden konnte.

In der COVID-19-Pandemie erfordert die dynamische Entwicklung eine kontinuierliche Bewertung der Lage. Als Basis dafür beschreiben *Halm et al.* in ihrem Beitrag die interne und externe Koordination und Kommunikation für das Lagemanagement des RKI und den regelmäßigen Austausch mit den zuständigen Behörden der Bundesländer. Dabei heben sie die Bedeutung einer guten Vorbereitung auf komplexe Geschehen hervor, um strukturelle und personelle Herausforderungen zu bewältigen. Anhand des Beispiels von Schleswig-Holstein kann nachvollzogen werden, wie die Bewertung der Lage auf Länderebene aussehen kann. Obwohl Schleswig-Holstein im Verlauf der Pandemie vergleichsweise geringe Inzidenzen aufwies, konnten *Rose et al.* anhand der Meldedaten erkennen, zu welchem Zeitpunkt welche Bevölkerungsgruppen besonders von der Pandemie betroffen waren.

Die Bewertung der Lage sollte als Basis für die Erstellung von Handlungsempfehlungen genutzt werden. Wie im Beitrag von *Grote et al.* dargelegt, konnte aufbauend auf den bestehenden Influenzapandemieplänen und den Erkenntnissen zu dem neuen Erreger in den ersten Monaten der COVID-19-Pandemie eine Multikomponentenstrategie entwickelt werden, bestehend aus bevölkerungsbasierten und individuellen infektionshygienischen Maßnahmen und ergänzt durch pharmakologische Maßnahmen. Von besonderer Bedeutung für die Bekämpfung der Pandemie sowie die Gewinnung von neuen Erkenntnissen zum Erreger ist die Untersuchung von Ausbrüchen durch die Gesundheitsämter. Das RKI unterstützt die Gesundheitsämter bei Bedarf beratend oder durch Feldteams vor Ort. *Alpers et al.* zählen in ihrem Beitrag zahlreiche Beispiele für diese Unterstützung in unterschiedlichsten Settings auf, die zum besseren Verständnis der Erregereigenschaften und zum Management beigetragen haben. Die Beispiele zeigen, wie durch die Untersuchungen epidemiologische Evidenz geschaffen werden konnte, welche wiederum als Grundlage für Empfehlungen herangezogen werden konnte.

In den letzten 3 Beiträgen werden einige durch die Pandemie entstandene Herausforderungen des ÖGD dargestellt. Beispielsweise hat der internationale Grenzverkehr dazu beigetragen, dass sich SARS-CoV‑2 initial schnell weltweit verbreiten konnte. In dem Beitrag von *Klein-Kampmann et al. *werden im Flug- und Schiffsverkehr vollzogene Maßnahmen, die damit verbundenen Schwierigkeiten und die daraus gewonnenen Erkenntnisse aus Sicht des ÖGD geschildert. Hervorzuheben ist dabei, dass eine Stärkung des ÖGD an den Grenzübergangsstellen sowie eine stärkere Digitalisierung für die Bewältigung der Aufgaben im Rahmen der Pandemie essenziell sind. Die besondere Belastung des ÖGD wird auch im Beitrag von *Horacek et al.* deutlich. Eine Befragung der Leitungen von Kinder- und Jugendgesundheitsdiensten (KJGD) in Nordrhein-Westfalen hat ergeben, dass das KJGD-Personal während der Pandemie fast ausschließlich im Infektionsschutz eingesetzt wurde und dadurch wichtige originäre Aufgaben wie etwa Schuleingangsuntersuchungen nicht erfüllt werden konnten. Zuletzt werden im Beitrag von *Gerlinger et al.* am Beispiel des ÖGD Gemeinsamkeiten und Unterschiede bei den Maßnahmen zur Pandemiebekämpfung in Schweden, Frankreich und Österreich aufgezählt. Obwohl sich die Struktur des ÖGD in diesen Ländern teilweise sehr unterscheidet, wird deutlich, dass in allen untersuchten Ländern der Verlauf der Pandemie und die Schutzmaßnahmen ähnlich waren. Der Schutz vulnerabler Gruppen wurde in allen Ländern von Beginn an verfolgt, ist aber nur unzureichend gelungen.

Im vorliegenden Themenheft wird deutlich, dass die COVID-19-Pandemie bisher in den Gesundheitsämtern, den Landesgesundheitsbehörden und dem Robert Koch-Institut zu einer beispiellosen Mobilisierung der vorhandenen Ressourcen geführt hat. Obwohl bereits Ende 2020 durch die Zulassung von hochwirksamen Impfstoffen die Hoffnung bestand, die Pandemie in absehbarer Zeit zu überwinden, bergen die neuen Virusvarianten die Gefahr von zahlreichen weiteren SARS-CoV-2-Infektionen, schweren Erkrankungen und Todesfällen [4]. Der ÖGD wird daher auch weiterhin stark gefordert sein. Die derzeitige Gesundheitskrise ist jedoch auch eine Chance, den durch jahrelange Einsparungen geschwächten ÖGD nachhaltig zu stärken und neu aufzustellen, etwa durch den „Pakt für den ÖGD“ [5].

## Footnotes

1 https://www.who.int/director-general/speeches/detail/who-director-general-s-opening-remarks-at-the-media-briefing-on-covid-19---11-march-2020.
